# Construction of Simple Sequence Repeat-Based Genetic Linkage Map and Identification of QTLs for Accumulation of Floral Volatiles in Lavender (*Lavandula angustifolia* Mill.)

**DOI:** 10.3390/ijms26083705

**Published:** 2025-04-14

**Authors:** Pavlina Georgieva, Krasimir Rusanov, Mila Rusanova, Meglena Kitanova, Ivan Atanassov

**Affiliations:** 1 Department of Agrobiotechnology, AgroBioInstitute, Agricultural Academy, 8 Dragan Tsankov Blvd., 1164 Sofia, Bulgaria; pavlina_vassileva@abv.bg (P.G.); krusanov@abv.bg (K.R.); milagradeva@abv.bg (M.R.); 2 Faculty of Biology, Sofia University, 8 Dragan Tsankov Blvd., 1164 Sofia, Bulgaria; m.kitanova@uni-sofia.bg; 3 Centre of Competence “Sustainable Utilization of Bio-Resources and Waste of Medicinal and Aromatic Plants for Innovative Bioactive Products” (BIORESOURCES BG), 1000 Sofia, Bulgaria

**Keywords:** lavender, *Lavandula angustifolia*, SSR markers, genetic linkage map, QTL mapping, floral volatile terpenoids

## Abstract

In spite of the increasing industrial cultivation of lavender (*Lavandula angustifolia* Mill.), no genetic linkage map and mapping of QTLs (quantitative trait locus) has been reported for *Lavandula* species. We present the development of a set of SSR (simple sequence repeat) markers and the first genetic linkage map of lavender following the genotyping of a segregating population obtained by the self-pollination of the industrial lavender variety Hemus. The resulting genetic map comprises 25 linkage groups (LGs) corresponding to the chromosome number of the lavender reference genome. The map includes 375 loci covering a total of 2631.57 centimorgan (cM). The average marker distance in the established map is 7.01 cM. The comparison of the map and reference genome sequence shows that LG maps cover an average of 82.6% of the chromosome sequences. The PCR amplification tests suggest that the developed SSR marker set possesses high intra-species (>93%) and inter-species (>78%) transferability. The QTL analysis employing the constructed map and gas chromatography/mass spectrometry (GC/MS) dataset of flower extracted volatiles resulted in the mapping of a total of 43 QTLs for the accumulation of 25 different floral volatiles. The comparison of the genome location of the QTLs and known biosynthetic genes suggests candidate genes for some QTLs.

## 1. Introduction

Lavender (*Lavandula angustifolia* Mill.), lavandin (*Lavandula × intermedia* Emeric ex Loisel.), and to some extent, spike lavender (*Lavandula latifolia* Medik.) are widely industrially cultivated members of the genus *Lavandula* worldwide [1,2,3,4,5]. The industrially cultivated lavender and lavandin are mainly used for the production of essential oil and, to a lesser extent, of concrete and hydrolate [2,3,6]. Due to its unique fine fragrance, lavender oil is widely used in perfumery, cosmetics, aromatherapy, and medicinal applications [7,8,9,10]. Cultivated lavandin provides a higher yield of essential oil per acre than lavender, but its sensory profile is less sophisticated and attractive, which assigns the use of lavandin oil mainly in the industrial perfumery. Bulgaria, France, and China have been the largest lavender oil suppliers in the world during the last decade [3,11], whereas the production of lavandin oil has been dominated by France [2,4]. The requirement that the produced essential oil must meet the ISO standards for lavender and lavandin oil has resulted in the growing demand for production and the use of high-quality planting material from superior vegetatively propagated varieties [2,11]. The increased use and demands of lavender and lavandin oil further attracts farmers and companies from various countries to expand the industrial cultivation and processing of lavender and lavandin, including the start of cultivation in new areas [6,11,12,13,14]. The expansion of lavender and lavandin farming areas is often related to their cultivation in soil and/or climatic conditions that significantly differ from the optimal for the varieties initially developed for the environmental conditions specific for the traditional areas of cultivation [13,15]. Often, such cultivation at the sub-optimal conditions of the available varieties results in large variations in flower and essential oil yields and quality, as well as significant economic losses. In addition, the introduction of new technologies for the mechanization of lavender and lavandin cultivation, processing, and extraction often requires the use of varieties suited for the application of that particular technology [6,16,17,18]. This suggests the need for the development of genomic resources and related molecular marker tools for better characterization of the available lavender and lavandin genetic resources. Expanding lavender cultivation in new areas, together with the market demands to increase the production of high-quality lavender oil, requires the prompt development of practical molecular tools for the identification of QTLs for important agronomic traits and essential oil quality and the application of marker-assisted selection for the efficient breeding of new elite varieties to meet the new challenges of the industry [2,11,19,20].

The biosynthesis and accumulation of volatile organic compounds in lavender and lavandin flowers were the subject of a number of studies, which resulted in the identification of a set of genes involved in particular biosynthetic steps, for example [21,22,23,24,25,26], or the regulation of these processes [27,28]. These studies were further supported by transcriptome sequencing [29,30,31] and the genome-wide characterization of gene families related to the terpenoid biosynthesis pathways [32,33,34], as well as by the reported nuclear genome sequence assemblies of three *L. angustifolia* varieties, including var. Maillette [35], var. Jingxun 2 [36], and the reference genome sequence of var. Munstead [37], accessible through the GenBank database. Accordingly, lavender was proposed as a model of aromatic and medicinal plants to study the biosynthesis of volatiles and the related ecology and evolution [15,31,38,39,40]. So far, the characterization of the genetic diversity and resources of *Lavandula* species has been the subject of few studies employing expressed sequence tags derived from simple sequence repeat (EST-SSR) markers [41], sequence-related amplified polymorphism (SRAP) markers [42], and random amplified polymorphic DNA (RAPD) markers [43].

Over the last three decades, the construction of genetic linkage maps and QTL analysis has been widely applied as an effective tool for the identification of loci for important agronomic traits and the direct application of the obtained results in marker-assisted selection programs in various crops, including medicinal and aromatic plants [44,45]. Although the construction of genetic maps increasingly involves the application of SNP-based markers, the genetic linkage maps based on SSR markers offer the attractive opportunity to be used as core genetic maps and directly applied for different genotypes of the species of interest, due to the high transferability of SSR markers. In spite of the large number of molecular genetics and genomic studies and related data in lavender and lavandin, surprisingly, no construction of a genetic linkage map has been reported for them, as well the identification and mapping of QTLs related to important traits. Moreover, the only SSR markers reported so far for *Lavandula* species is a set of EST-SSR markers [41], which are not sufficient for genome mapping studies and QTL analysis.

In the present study, we report the development of an extended set of SSR markers for lavender, the construction of the first SSR-based genetic linkage map for this species, and the identification of QTLs for the accumulation of floral volatiles, following the characterization of an F2 segregating population obtained by the self-pollination of the industrial lavender variety, Hemus.

## 2. Results and Discussion

### 2.1. Development of an F2 Segregating Population by Self-Pollination of L. angustifolia var. Hemus

Variety Hemus of *L. angustifolia* was established in 1974 and, over the years, has become one of the most widely industrially cultivated lavender varieties in Bulgaria. It is suitable for mechanized harvesting, tolerates cultivation in different geographic regions in the country [46], and provides high-quality distilled lavender oil. For some period of time, var. Hemus was also used as a state standard for the evaluation of the performance of newly developed lavender varieties. Since its initial development, var. Hemus has been propagated vegetatively, both for maintenance in genetic resources collections and for the production of planting material. Considering all this, var. Hemus was selected for the development of a segregating population and genetic linkage map of lavender. Our early attempt to develop F2 segregating population through the spontaneous or mechanical self-pollination of a single var. Hemus plant was not successful. No seed formation was observed after covering the entire plant with a cage of fine mesh for the isolation of insects, which supports the recently reported lack of spontaneous self-pollination in lavender varieties cultivated in the country [47]. Therefore, to generate a larger seed set after self-pollination, a single var. Hemus plant was grown in an area where no other *L. angustifolia* plants were detected. As a result, over 1200 seeds were collected from a single var. Hemus plant during the 2020 flowering season due to the pollinator-mediated self-pollination. The germination and planting of these seeds resulted in the generation of a segregating population of a thousand plants. The SSR marker genotyping of 110 randomly chosen plants from this population confirms that they result from the self-pollination of the cultivated var. Hemus plant. Accordingly, 95 of these tested plants were selected as a mapping population. The results demonstrated that such an approach, or growing plants in insect-restrained rooms and the use of dedicated greenhouse insects as pollinators, could be applied for the generation of self-pollinated seeds from the plants of other species from the *Lamiaceae* family, already reported as difficult for self-pollination [48,49].

### 2.2. SSR Marker Development

Three groups of primer pairs were tested for the amplification of genomic regions containing SSR sequences and the development of a large set of SSR markers for genotyping the segregating population, including 471 next generation sequencing-based simple sequence repeat (NGS-SSR) primer pairs determined after the analysis of Illumina MiSeq-generated genomic sequences of var. Hemus, 170 genome-based simple sequence repeat (GEN-SSR) primer pairs designed after the analysis of the reference genome sequence of var. Munstead retrieved from GenBank, and 22 EST-SSR primer pairs reported by Adal et al. [41]. The testing included two rounds, first for the successful amplification of the target SSR region from var. Hemus and the production of a distinct profile of the amplified DNA fragments and the second round for generation of segregating alleles after the analysis of eight plants from the segregating population. The results of the tests are demonstrated in Table 1 and show that testing the NGS-SSR group was the most efficient for the development of polymorphic SSR markers. The testing of markers from the EST-SSR group resulted in the identification of a significantly lower portion of polymorphic markers, which supports the higher efficiency and informativeness of the genomic SSRs compared to EST-derived SSR markers also observed in other studies [50,51]. The testing of SRAP markers showed that this type of markers could be readily applied for the simultaneous genotyping of a large number of loci, due to the higher number of polymorphic alleles, which could be detected and analyzed by a single marker/primer pair. Thus, SRAP markers could be efficiently applied to increase the marker density of SSR-developed genetic maps.

### 2.3. Linkage Map Construction

**Figure 1:**
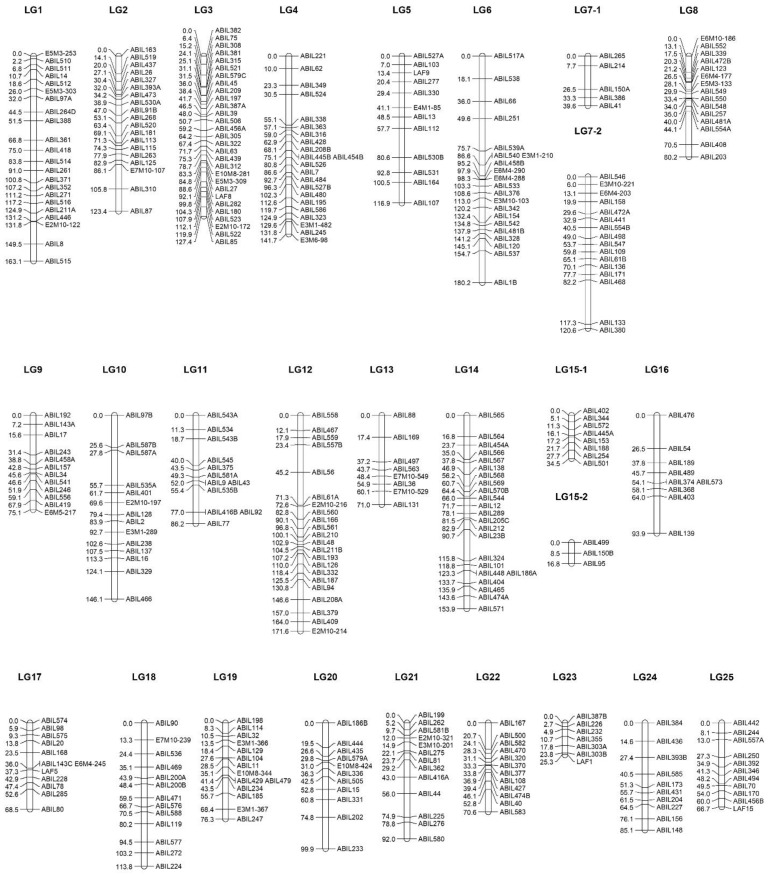
Genetic linkage map of *L. angustifolia* var. Hemus.

A total of 411 loci were analyzed for linkage mapping, including 377 loci obtained through genotyping with three groups of SSR markers and 34 loci identified through analysis with SRAP markers. The first round of map construction involved genotyping the segregating population with five previously reported EST-SSR markers [41] and 255 NGS-SSR markers, developed after the analysis and testing of SSR regions in genomic sequences of *L. angustifolia* var. Hemus. This first version of the map was developed shortly before the publication in GenBank of the reference genome sequence of *L. angustifolia* var. Munstead. Using the latter BLAST+ 2.16.0 search with the primer and SSR region sequences in the reference genome sequence was used to affiliate the constructed LG maps to the corresponding reference chromosome sequences and for determining the physical position of the mapped SSR loci. The obtained data were used to proceed in a second round of map expansion through the identification, selection, and testing of GEN-SSR primer pairs for the amplification of SSR regions located in the chromosome regions outside of the range of the corresponding linkage groups or located in the low marker density regions of the map. All this resulted in the mapping of additional 90 loci after analysis with 79 GEN-SSR markers. Finally, the segregating population was genotyped with 11 SRAP markers, which resulted in the mapping of 34 SRAP loci. The overall data analysis and quality check of the obtained genetic map resulted in the identification of a total of 25 linkage groups using an LOD score of 5.0, which corresponds to the number of assembled chromosome sequences in the reference genome, as well as to the chromosome number of var. Hemus used for the development of the segregating population. The further data analysis at a higher stringency showed the preservation of the formed LGs at an LOD score of 7.0, as only two linkage groups, LG7 and LG15, were resolved at two sub-groups each, Figure 1. The main parameters of the map are shown in Table 2, and details for the position of each locus are presented in Table S3, Supplementary Materials. The constructed genetic linkage map of *L. angustifolia* var. Hemus contains 375 loci, covering a total of 2631.57 cM. The average marker distance in the map is 7.01 cM. The number of markers on each LG ranged from 7 (LG 23) to 29 (LG 3), with an average number of 15, and the length per LG ranged from 25.23 cM (LG 25) to 180.24 cM (LG 6), with an average length of 105.26 cM. A total of 36 analyzed loci were not included in the map. Of them, 14 loci had no distinct affiliation to the established LGs, Table S3. The other 22 loci initially affiliated to different LGs were excluded from the final map, since they showed segregation distortion, and their inclusion resulted in significant changes in the marker order in the maps of the corresponding LG, Table S3. The appearance of loci with segregation distortion, which significantly changed the established map after their inclusion, was reported for different plant species, for example [52,53,54,55,56], and could be expected for continuously vegetatively propagated lavender varieties, like var. Hemus. The performed BLAST search revealed that the sequences of two NGS-SSR markers, ABIL245 (LG4) and ABIL247 (LG19), were allocated on two scaffold sequences of the reference genome, scaffold 293 (acc. JAPVEC010000093) and scaffold 515 (acc. JAPVEC010000026), which were not included into the chromosome sequence assemblies, Table S3. In reverse, the genotyping of the GEN-SSR marker ABIL587 targeting an SSR region on scaffold 246 (acc. JAPVEC010000206) was affiliated to a locus on LG18, whereas the GEN-SSR marker ABIL588 targeting an SSR region on scaffold 229 (acc. JAPVEC010000199) corresponded to loci on LG10 (ABIL588A and ABIL588B) and LG23 (ABIL587C and ABIL588D), Table S3. Accordingly, the established genetic map and additional genotyping of selected SSR markers could be further applied to clear some ambiguities in the current reference chromosome assembly and affiliate some of the scaffolds to particular chromosomes. The use of high-density genetic maps for improving reference genome assemblies has been reported in other plant species. [57,58,59].

The BLAST search determined the positions of SSR sequences in the reference genome, and a comparison with the respective SSR loci positions in the established genetic map shows that the order of SSR loci in the LGs well resembles those predicted from the corresponding chromosome sequence assemblies, Table S3. The affiliation of the sub-linkage groups LG7-1, LG7-2, LG15-1, and LG15-2 to the larger groups LG7 and LG15 was confirmed by their affiliation to the sequences of chromosome 7 and 15, Table S3. Closer observation of the marker order in the linkage groups and the location of the corresponding SSR sequences in the chromosomes show that the positions of less than 5% of the mapped SSR markers differ from the order predicted based on the reference genome. The reasons for these differences are not clear and could be a result of local differences in the genomic sequences or chromosome rearrangements in the genome of var. Hemus or/and var. Munstead, local map disorders due to insufficient marker saturation, or even in local genome sequence disorders due to local irregularities of the genome sequence assembly. Further saturation of the map, including the regions surrounding these markers, will provide valuable information to clear these issues. Such local saturation and improvement of the map could be carried out in a genome sequence guided manner, similar to the development of GEN-SSR markers in the present study. The overall comparison of the established LG maps and corresponding positions of the markers in the reference chromosome sequences makes it possible to estimate the current genome coverage of the map. The comparison shows that the genetic map covers between 37.2% and 99.4% of the reference chromosome sequences, with an average coverage of 82.6% +/− 16.7% per chromosome, Table 2.

### 2.4. SSR Marker Transferability

Intra- and inter-species transferability of the employed SSR markers is essential for the various applications of sub-sets of these SSR markers, as well as of the established genetic linkage map and the construction of an SSR marker-anchored reference genetic map of lavender and *Lavandula* sp. To evaluate the transferability of the SSR markers applied in the established map, the SSR primer pairs were tested for the PCR amplification of genomic DNAs isolated from *L. angustifolia* var. Hidcote Blue, two *L. latifolia* accessions, and the intersectional hybrid *L. × heterophylla* var. Big Boy James. A summary of the results is presented in Table 3 and described in detail in Table S4, Supplementary Materials. The obtained results show the high transferability of the majority of SSR markers used for the development of the genetic map, as above 93% of the tested SSR primer pairs show positive PCR amplification for the *L. angustifolia* garden variety Hidcote Blue, and above 78% of the SSR markers exhibit the positive PCR amplification of DNA from other *Lavandula* species. The observed high intra- and inter-species marker transferability suggests that the developed marker set could be efficiently employed for the evaluation of the genetic diversity of natural populations and genetic resources collections of *Lavandula* species, as well as being further applied for the development of an integrated genetic map of *L. angustifolia* and SSR marker-anchored genetic maps in the genus *Lavandula*. Such SSR marker-anchored genetic maps have been successfully and increasingly applied in the modern plant breeding of various cultivated plant species [60,61,62].

### 2.5. Variations of Volatile Contents in the Flowers of Plants from the Segregating Population

Correct comparative analysis of aroma traits is a challenging task, since the accumulation of volatiles is influenced by a variety of factors. Several studies have reported differential biosynthesis and the accumulation of various volatiles during the development of lavender flowers and inflorescences and during different daytime periods [26,33,63,64]. Accordingly, for the GC/MA analysis of the flower volatile composition in this study, we employed a simple procedure for sample preparation involving organic solvent extraction of a fixed number of florets at a stage of development corresponding to fully developed petals [65]. This sample preparation allowed for the collection of flower samples from the plants of the entire segregating population within two hours, thus avoiding variations due to flower developmental and environmental factors. The GC/MS analysis resulted in the identification and quantification of 34 compounds, Table 4. Further comparison of the obtained data showed large variations in the composition of the analyzed floral volatiles among the plants from the segregation population. This was well demonstrated by the determined minimal and maximal content of each analyzed volatile scored for the tested plants from the segregating population, as well as from the calculated ratios of the minimal and maximal content to the average content for the entire population, Table 4. Accordingly, for several of the analyzed volatiles, these calculated minimal and maximal ratios ranged from zero to over 300–500% of the average for the population, Table 4.

### 2.6. Mapping of QTLs Related to the Accumulation of Flower Volatiles in L. angustifolia var. Hemus

Mapping QTLs related to the accumulation of flower volatiles is an essential step for marker-assisted selection and the breeding of elite lavender lines and varieties for the production of high-quality essential oil. An early version of the constructed SSR map was already successfully applied for the identification of a QTL, controlling the ratio of linalool to linalyl acetate in the flowers of *L. angustifolia* var. Hemus [66]. Here, we performed QTL analysis for the accumulation of volatiles using the dataset from the GC/MS analysis of flower volatiles and the genetic linkage map developed in this study. The QTL analysis resulted in the identification and mapping of a total of 43 QTLs for 25 volatile compounds. No significant QTLs were identified for nine compounds after applying genome-wide significance threshold based on a 5% experimental error rate. The identified QTLs were distributed across 12 linkage groups, with an LOD score ranging from 4.03 to 17.35, Table 5. The number of QTLs per trait ranged from 1 to 4. The proportion of volatile content variance explained (PVE) by the identified QTLs ranged from 20.1% to 62.7%.

The closer observation of the markers surrounding the identified QTLs show full overlap of the genomic regions of some of the QTLs related to different compounds, suggesting their affiliation to common biosynthetic pathway and even common genetic background, Table 6. To examine the possible genetic and biosynthetic background of the identified QTLs, their genome locations were compared with the locations of the sequences homologous to earlier identified genes from the metabolic pathways related to the biosynthesis of corresponding volatile compounds. The results demonstrated that a large portion of the genomic regions of the identified QTLs also included the genomic sequences of genes involved in the biosynthesis of the corresponding compounds, which provide further support for the significance and genetic background of these QTLs, Table 6. For example, the 622 kbp genomic region of q13-8.1 of linalool overlap with q14-8.1 of linalyl acetate and also include the genomic sequence homologous to an AAT4 gene, which is a member of the BAHD acyltransferase family and was reported to convert lavandulol to lavandulyl acetate [67]. In addition, some of the regions related to overlapping QTLs include a cluster of genomic sequences homologous to known genes involved in the biosynthesis of the QTL compounds. For example, the 3,6 Mbp genomic region overlapping q23-5.1 (γ-Cadinene) and q25-5.1 (tau-Cadinol) include four sequences homologous to the LaCADS gene, which catalyzes the production of various by-products, including γ-cadinene and tau-Cadinol. The presence in the identified QTL region of a cluster of candidate genes raises the question about the function and impact of the individual members of the cluster to the biosynthesis and accumulation of the QTL volatiles. On the other hand, it offers the opportunity for the direct application of the SSR markers surrounding the QTL of interest in breeding programs just accounting on the overall impact of the QTL.

## 3. Discussion

Here, we reported the construction of the first genetic linkage map of lavender based on SSR markers. The comparison of genetic and sequence data shows that the map order of the SSR loci corresponds well to their location in the reference genome sequence. This provides an opportunity for efficient further increase in the density of the map, including directed local saturation through the target development of additional SSR markers in the region of interest based on genome sequence data. The demonstrated high intra- and inter-species transferability of the SSR markers facilitate further wider and efficient applications of the SSR markers and established maps in various genetic studies, including the characterization of lavender genetic resources and marker-assisted selection. The performed QTL analysis demonstrates that, in spite of the moderate density of the constructed genetic linkage map, it can be efficiently employed for the identification of QTLs related to the accumulation of volatile compounds and other important agronomic traits in lavender. Moreover, the codominant nature of the SSR markers, together with their high transferability, provide an attractive opportunity for the straightforward and low-cost testing of diverse lavender populations for the presence and impact of the already identified QTLs, as well as for testing the possible impact of the alleles of known genes. Such testing could be easily realized simply by SSR genotyping and the determination of the allele configurations of two SSR loci surrounding the region of interest and a comparison of the parameters of the studied trait for the groups of plants with the same allele configurations for the tested alleles. Generally, the first round of such testing does not necessarily require a high density of the map, and meaningful results could be obtained, even when the target genomic region is situated between two rather widely located SSR loci. Besides testing the impact of the already identified QTLs or genes of interest, such SSR analysis could also be used for the identification of plants with a recombination in the target region and the subsequent identification of candidate genes related to the studied QTL. The last could be carried out through marker walking within the region of interest via the selection and application of new SSR markers from the reference genome sequence, as described for GEN-SSR markers. Considering the increasing need from the application of marker-assisted selection in lavender breeding, we continue with the efforts to increase the density of the current genetic map of var. Hemus and the further construction of an integrated genetic map of the industrially cultivated lavender varieties.

## 4. Materials and Methods

### 4.1. Plant Material

*L. angustifolia* var. Hemus plant used for the development of the F2 segregating population was obtained from the Institute of Roses and Aromatic Plants/IRAP/, Kazanlak, Bulgaria. Variety Hemus was established by IRAP breeders in 1974, following the pick-up and clonal selection of individual plants from the seed population. Since then, var. Hemus has been vegetatively propagated for the production of planting material and genetic resource collection. For the development of the segregating population, a single plant of var. Hemus was cultivated in the area of the village of Zagore, Thracian lowland, Bulgaria, and seeds were collected after the 2020 flowering period. No other lavender plants were detected to grow in the same area during this period. The seeds were stratified for 12 weeks at 4 °C and germinated in a tray on planting soil. The obtained seedlings were transferred in pots, grown in the greenhouse for 10 weeks, and cultivated in the experimental field of the Agrobioinstitute (ABI), Kostinbrod, Bulgaria. Var. Hemus (mother plant), and 95 plants from segregating population were labelled and further sampled. The plants of *L. angustifolia* var. Hidcote Blue, *L. latifolia*, and the intersectional hybrid *L. × heterophylla* ’Big Boy James’ were purchased from Bastin Nursery (Kwekerij Bastin, Aalbeek, the Netherlands). The *L. latifolia* (Ll_abi2) plant was grown and selected from seeds purchased from WeberSeeds Botany Ethnobotany, the Netherlands. The plants were grown in pots in the greenhouse of ABI, Kostinbrod, Bulgaria.

### 4.2. Genomic DNA Isolation

About 20–25 young leaves from every plant were placed in 15 mL plastic containers, immediately frozen in liquid nitrogen and stored at −80 °C. The frozen leaf samples were ground to a fine powder by using the Qiagen TissueLyser II Mill (QIAGEN AG, Steinhausen, Switzerland) at 30 Hz for 2 min. Genomic DNA was purified according to the cetyltrimethylammonium bromide (CTAB) protocol [72]. Genomic DNA concentration was measured spectrophotometrically by using Nanodrop 2000 (Thermo Fisher Scientific, Waltham, MA, USA) and diluted to a final concentration of 25 ng/μL with Type I ultrapure water.

### 4.3. Next Generation Sequencing, SSR Identification, and Primer Design

Microsatellite sequences from *L. angustifolia* var. Hemus were identified as a service by Ecogenics GmbH, Switzerland following the NGS sequencing of a genomic DNA sample isolated from the var. Hemus plant used for the development of the segregating population. The Illumina TruSeq nano DNA library was sequenced by using MiSeq Reagent Nano Kit v2 and Illumina MiSeq sequencing platform (Illumina Inc., San Diego, CA, USA). The paired-end reads were passed through Illumina’s chastity filter and were further subject to de-multiplexing and trimming of Illumina adaptor residuals. The quality of the surviving reads was checked with FastQC v0.11.8 [73]. The paired-end reads were next quality filtered and merged with USEARCH v11.0.667 [74] to reform in silico the sequenced molecules. The merged reads were screened with the software Tandem Repeats Finder, v4.09 [75]. Merged reads containing SSR region with at least six repeat units of tri- or a tetra-nucleotide or at least ten repeat units a dinucleotide of were selected. Primer 3 [76,77] software was used for primer design. Raw NGS sequences can be accessed at the NCBI Sequence Read Archive under accession number PRJNA1207064.

### 4.4. SSR Identification After Search of Reference Genome Sequence and Primer Design

The reference genome sequence of *L. angustifolia* var. Munstead was downloaded from the NCBI website https://www.ncbi.nlm.nih.gov/datasets/genome/?taxon=39169, GenBank acc. No GCA_028984105.1 (accessed on 1 June 2024) [37,78]. The genome sequence includes 795,075,733 bp chromosome-scale assembly, representing 25 chromosomes with a N50 scaffold length of 31,371,815 bp [37]. The Krait Microsatellite Identification and Primer Design tool (v1.5.1) [79] was used for the search of the reference genome sequence for the presence of SSR motifs. The following minimal parameters for SSR identification were applied: 12 for mononucleotides, 7 for dinucleotides, 5 for trinucleotides, 4 for tetranucleotides, 4 for pentanucleotides, and 4 for hexanucleotides. The primer pairs flanking the selected SSRs were designed to amplify predicted PCR products ranging from 100 bp to 300 bp. In addition to the primers for SSR regions identified from NGS and genome sequence data, five more primer pairs for the PCR amplification of EST-SSR loci LAF1, LAF5, LAF8, LAF9, and LAF15, described by Adal et al. [41], were used within the study. All primers were synthesized by Macrogen Europe BV, the Netherlands. The primer sequences of primer pairs used for genotyping plants from the segregating population are presented in Table S1, Supplementary Materials. The theoretical positions of the applied SSR markers were determined after the BLAST search of the reference genome sequence of *L. angustifolia* var. Munstead by using the primer sequences and the sequence of the SSR genome region obtained from NGS data for var. Hemus.

### 4.5. PCR Amplification of SSR Regions

The PCR amplifications of SSR regions from plant genomic DNA, for testing the transferability of SSR markers or for analysis by the Agilent 5200 Fragment Analyzer System, were performed in a volume of 16 µL, including 1 µL of forward primer (10 pmol/µL), 1 µL of reverse primer (10 pmol/µL), 8 µL of 2× MyTaqTM Mix (Meridian Bioscience, Cincinnati, OH, USA), 4.7 µL ultra-pure water, and 1.3 µL genomic DNA (25 ng/µL). The following PCR conditions were used: 95 °C for 3 min followed by 33 cycles of 95 °C for 15 s, 57 °C for 30 s, 72 °C for 30 s, and a final elongation at 72 °C for 10 min. For testing the SSR marker transferability, the PCR products were resolved after electrophoresis in 1.8% agarose gels and observed under UV light. For fragment analysis and plant genotyping, the PCR products were subject to analysis by the Agilent 5200 Fragment Analyzer System (Agilent Technologies, Inc., Santa Clara, CA, USA) using the dsDNA 905 Reagent Kit (1–500 bp).

### 4.6. PCR Amplification of SSR Regions with Tailed Primers

The PCR amplifications of SSR regions from plant genomic DNA, subject of fragment analysis by a fluorescent capillary sequencer, were carried out using the designed reverse and 5′-tailed forward primers. Five different types of tails [80,81] were used, including M13 tail (5′-TAAAACGACGGCCAGT), A tail (5′-GCCTCCCTCGCGCCA), B tail (5′-GCCTTGCCAGCCCGC), C tail (5′-CAGGACCAGGCTACCGTG), and D tail (5′-CGGAGAGCCGAGAGGTG). The tails used for the 5′-tailing of the forward primer were M13 tail when the Tm of the reverse primer was ≤58 °C, C and D tails when the Tm of the reverse primer were 58 °C < Tm ≤ 62 °C, and A and B tails when the Tm of the reverse primer were >62 °C. The tails were synthesized and 5′-labeled with FAM, JOE, ROX, and TAMRA dyes, as a service by Macrogen Europe BV, the Netherlands. All tails and primer combinations for the used primer pairs are presented in Table S1, Supplementary Materials. Based on the specific tail used, the annealing temperatures (Ta) of the PCR reactions were 54 °C for M13 tail, 57 °C for C and D tails, and 59 °C for A and B tails. The PCR reactions were performed in a volume of 16 µL, containing 0.8 µL of 5′-tailed forward primer (3 pmol/µL), 1 µL of 5′-labeled tail primer (10 pmol/µL), 1 µL of reverse primer (10 pmol/µL), 8 µL 2× MyTaqTM Mix (Meridian Bioscience), 4 µL ultra-pure water, and 1.2 µL genomic DNA (25 ng/µL). The following PCR conditions were used: 95 °C for 3 min followed by 33 cycles of 95 °C for 15 s, Ta for 30 s (Ta is according to the type of tail used, described above), 72 °C for 30 s, and a final elongation at 72 °C for 10 min. The PCR-amplified DNAs were further subjected to fragment analysis using ABI 3130 Genetic Analyzer (Thermo Fisher Scientific, Waltham, MA, USA).

### 4.7. PCR Amplification of SRAP Fragments

In addition to the SSR marker genotyping, eleven SRAP primer pairs were used for the amplification of SRAP fragments from the genomic DNA of *L. angustifolia* var. Hemus and the plants of the analyzed segregating population. The ME and EM type of SRAP primers were designed according to Li and Quiros [82] and are shown in Table S2, Supplementary Materials. The PCR reactions were carried out in a volume of 20 µL, containing 1.5 µL of DNA template (25 ng/µL), 1 µL of 5′-labeled forward EM primer (10 pmol/µL), 1 µL of reverse ME primer (10 pmol/µL), 10 µL 2× MyTaq HS Mix (Meridian Bioscience), and 6.5 µL ultra-pure water. Samples were PCR-amplified using the following thermal profile: 5 min at 94 °C; 3 cycles of 1 min at 94 °C, 1 min at 35 °C, and 1 min at 72 °C; 35 cycles of 1 min at 94 °C, 1 min at 50 °C, and 1 min at 72 °C; and a final elongation step of 3 min at 72 °C. The forward ME primers were 5′-end labelled with FAM. The PCR-amplified DNAs were further subjected to fragment analysis using ABI 3130 Genetic Analyzer (Thermo Fisher Scientific, Waltham, MA, USA).

### 4.8. SSR and SRAP Fragment Analysis

ABI 3130 Genetic Analyzer with 36 cm long capillaries, Pop-7 polymer, and GeneScan^TM^ 500 LIZ^TM^ size standard (all from Thermo Fisher Scientific, Waltham, MA, USA) were used for fragment analysis of the amplified SSR an SRAP fragments, as described earlier [42,50]. The fragment sizing was carried out by using GeneMapper 4.0 (Thermo Fisher Scientific, Waltham, MA, USA). The allele combination for each SSR locus and analyzed plants was determined following a comparison with the alleles of the mother *L. angustifolia* var. Hemus plant. The SRAP fragment analysis was carried out for fragments in the range of 60–600 bp. All SRAP fragments showing a distinct presence or absence in the SRAP patterns of the analyzed plants from the segregating population were scored. Each of these SRAP fragments was considered a dominant allele of a separate locus, and the obtained data were used in the linkage analysis.

### 4.9. Linkage Analysis and Genetic Map Construction

JoinMap 5 [83] was used for linkage analysis. The grouping was carried out by using the maximum likelihood (ML) mapping algorithm with the Haldane mapping function. The markers were grouped into linkage groups using an LOD score of 5.0 or higher. The values of nearest neighbor (NN) fit and NN stress from the Fit & Stress tabsheet and −log10P values from the Genotype Probabilities: Locus Means tabsheet were used to inspect the quality of the established map. Markers related to poor map quality criteria were excluded and after that re-introduced one by one, and the mapping analysis was performed again. If the re-introduction of a marker confirmed its poor fit, the marker was removed from further analysis until a good quality map was established. The obtained order of SSR markers in each linkage group was compared with their theoretical position in the reference genome sequence. When the order of SSR markers in the linkage groups differed from the order of their theoretical position in the corresponding chromosome of the reference genome sequence, the possible change in their order was tested through the evaluation of the map quality after using the fixed-order input in JoinMap 5 and observation of the map quality criteria (high NN fit, NN stress, or –log10P values). When the fixed order did not result in the deterioration of the map quality, it was considered to be a genetic map of the corresponding linkage group. The genetic maps were drawn with Map-Chart v2.32 [84].

### 4.10. Sample Preparation and GC/MS Analysis of Floral Volatiles

The preparation of extracts for the GC/MS analysis of floral volatiles was performed as described in Zagorcheva et al. [65], after collecting florets from two-year-old plants of the segregating population. Thirty florets with fully open petals, corresponding to stage 3 described by Guitton et al. [26], were collected in a 10 mL headspace glass vial. After the addition of 3 mL of hexane (Sigma-Aldrich, St. Louis, MO, USA) and 200 mg of anhydrous sodium sulfate (Sigma-Aldrich, St. Louis, MO, USA), the glass vials were crimp-capped and kept at 4 °C until analysis. Prior to analysis, the glass vials were vortexed at 1000 rpm on a VWR Multitube Vortex Mixer (VWR, Radnor, PA, USA) for 30 min. For each plant, one sample was analyzed in one replicate. A total of 1 mL of the extract was transferred to a 2 mL GC vial and subjected to GC/MS analysis on an Agilent 8890/5977B GC/MS system, as described earlier [65]. Briefly, the compounds were separated on a polar HP-INNOWax column (Agilent Santa Clara, CA, USA), 30 m × 0.25 mm, film thickness 0.25 μm, containing PEG as a stationary phase. Carrier gas was helium (purity 99.999%) at a flow rate of 0.8 mL/min. The injector temperature was 250 °C, using the splitless injection of 1 μL per sample. The oven program was as follows: 65 °C to 170 °C at 2 °C/min, then 60 °C/min to 240 °C, hold for 15 min. The mass selective detector was operated at a transfer line temperature of 250 °C and electron impact ionization voltage of 70 eV. The C10-C40 alkane mixture (Sigma-Aldrich, St. Louis, MO, USA) was used for calculating the Kovats retention index of each chromatographic peak using the AMDIS 2.73 software (National Institute of Standards and Technology (NIST), Gaithersburg, MD, USA). Individual compounds were identified based on their MS spectrum using the NIST 2007 mass spectral library (National Institute of Standards and Technology (NIST), Gaithersburg, MD, USA) as well as based on their Kovats retention index and literature data. The relative content of the analyzed compounds was determined as percentage of the total area of the chromatogram using Agilent Productivity Chemstation ver. F.01.03.2365 (Agilent, Santa Clara, CA, USA). Analysis of variance (ANOVA) for significance between means was performed using SPSS v. 26 (SPSS Inc, Chicago, IL, USA).

### 4.11. QTL Mapping

QTL analysis was performed using the interval mapping method implemented in MapQTL ver. 6.0 (Kyazma, Wageningen, The Netherlands). The significant QTLs were identified using a genome-wide LOD threshold based on a 5% experimental error rate, which was determined through 1000 permutations. The identified QTLs were named by combining the name of the trait and the chromosome number.

## 5. Conclusions

In this study, an extended set of SSR markers were developed and applied for the construction of the first genetic linkage map of lavender, using a segregating population obtained by the self-pollination of the industrial lavender variety Hemus. The SSR markers showed high intra- and inter-species transferability, allowing for their efficient application in studies including the characterization of the genetic resources and mapping studies in *Lavandula* sp. The constructed map was successfully employed for the identification and mapping of QTLs related to the accumulation of volatile terpenoids in the flowers of plants from the segregating population. The search of the lavender reference genome shows that the genomic regions of part of the identified QTL also include sequences homologous to genes involved in the biosynthesis of the QTL compounds, which pointed out candidate genes for further allele testing. The developed set of SSR markers and the constructed genetic map lay the groundwork for subsequent marker-assisted breeding in lavender. However, the further routine application of the markers employed in the map requires an additional increase in the density of the map and the construction of an integrated genetic map of the main industrially cultivated lavender varieties. The further dissection of the QTL regions of interest through marker walking and development and the application of markers tightly linked to the candidate genes will both increase the efficiency of marker-assisted selection and gain new knowledge on the functionality of the corresponding genes and their alleles.

## Figures and Tables

**Table 1:** Development and selection of polymorphic SSR and SRAP markers.

Type of the Tested Markers	Total Number of Tested Markers	Markers Showing Positive Amplification and Distinct Pattern (% from the Total Number of Tested Markers)	Number of Polymorphic Markers (% from the Total Number of Tested Markers) [% from the Markers with Positive Amplification]	Total Number of Loci Identified (Average Number of Detected Loci per Marker)
NGS-SSR	471	442 (93.8%)	255 (54.1%) [57.7%]	282 (1.11)
GEN-SSR	170	154 (90.6%)	79 (46.5%) [51.3%]	90 (1.14)
EST-SSR	22	17 (77.3%)	5 (22.7%) [29.4%]	5 (1.0)
SRAP	11	11 (100%)	11 (100%) [100%]	33 (3.0)
Total for the SSR markers	663	613	339	377

**Table 2:** Summary of the *L. angustifolia* var. Hemus genetic linkage map and location of the loci on the reference genome sequence.

Linkage Group	Number of Loci **	Map Length (cM)	Map Density (Loci/cM)	Largest Gap (cM)	Chromosome Sequence (Mbp)	Foremost Position of the Loci (Mbp)	Rearmost Position of the Loci (Mbp)	Coverage of the Chromosome (%)
LG1	22	154.23	7.01	17.69	43.13	1.64	40.38	89.8%
LG2	19	123.38	6.49	19.68	40.25	1.08	39.51	95.5%
LG3	29	127.30	4.39	8.93	39.13	1.54	39.09	96.0%
LG4	22	141.74	6.44	24.50	37.48	0.30	34.65	91.6%
LG5	12	116.89	9.74	22.92	35.70	1.86	34.68	91.9%
LG6	21	180.24	8.58	26.06	35.32	0.08	35.18	99.4%
LG7 *	21	160.20	7.63	18.83	34.57	0.05	34.21	98.8%
LG8	15	80.21	5.35	26.38	33.58	1.31	33.47	95.8%
LG9	12	75.12	6.26	15.83	33.03	10.06	30.87	63.0%
LG10	14	146.12	10.44	27.91	32.61	0.14	32.21	98.3%
LG11	12	86.21	7.18	21.59	31.76	0.78	31.00	95.2%
LG12	22	171.62	7.80	26.10	31.37	0.02	26.38	84.0%
LG13	8	71.03	8.88	19.81	30.09	7.42	26.93	64.8%
LG14	23	153.85	6.69	11.26	29.44	0.25	28.75	96.8%
LG15 *	11	51.39	4.67	8.46	28.69	1.16	27.98	93.5%
LG16	9	93.88	10.43	29.89	28.47	1.04	15.06	49.2%
LG17	12	68.54	5.71	15.70	27.94	2.54	22.52	71.5%
LG18	13	113.77	8.75	14.26	27.54	2.61	27.51	90.4%
LG19	14	76.30	5.45	12.75	27.40	0.11	19.59	71.1%
LG20	11	99.86	9.08	25.05	27.08	4.13	26.87	84.0%
LG21	13	92.03	7.08	18.86	27.05	5.20	26.54	78.9%
LG22	12	70.64	5.89	20.45	26.81	0.59	25.15	91.6%
LG23	7	25.23	3.60	7.07	26.63	14.18	24.09	37.2%
LG24	10	85.09	8.51	14.64	23.28	1.45	17.98	71.0%
LG25	11	66.70	6.06	14.25	22.94	1.37	16.55	66.2%
Minimal value for LG	7	25.23	3.60					37.2%
Maximal value for LG	29	180.24	10.44					99.4%
Average value per LG	15 +/−5.67	105.26 +/−40.73						82.6% +/−16.7%
Total value for the map	375	2631.57	7.01					

* LG7 and LG15 present a summary of the data for the corresponding sub-linkage groups (LG7-1; LG7-2; LG15-1; LG15-2). ** not including 36 analyzed loci, among them 14 loci for which no affiliation to a particular LG was found and 22 loci showing segregation distortion and affiliated to different LGs, of which inclusion resulted in significant changes in the LG map.

**Table 3:** SSR marker transferability.

PCR Amplification (*)	Results of SSR Amplification from DNA of the Tested Plants **			
	*L. angustifolia* var. Hidcote Blue	*L. latifolia* Ll_abi2	*L. latifolia* Bastin Nursery	*L. × heterophylla* var. Big Boy James
(+)	291	243	172	251
	93.9%	78.4%	78.5%	81.0%
(w)	10	15	7	10
	3.2%	4.8%	3.2%	3.2%
(−)	9	52	40	49
	2.9%	16.8%	18.3%	15.8%
Total number of tested SSRs	310	310	219	310

* (+) distinct PCR amplification similar to that of *L. angustifolia* var. Hemus, used as positive control; (w) weak PCR amplification; (−) no PCR amplification observed. ** percentage from the total number of tested plants.

**Table 4:** Results from GC/MS analysis of the composition of volatiles in the flowers of plants from the segregating population.

No	Compound	RT(s)	RI	Average * +/− Stdev (%)	Minimal * Content (%)	Maximal * Content (%)	Min/ Average ** (%)	Max/ Average ** (%)
1	Toluene	527.8	1039.0	0.329 +/− 0.117	0.007	0.778	2.0	236.8
2	Camphene	563.2	1055.2	0.131 +/− 0.079	0.027	0.416	20.8	317.2
3	β-Pinene	657.8	1098.8	0.045 +/− 0.031	0.007	0.186	16.0	411.5
4	Sabinene	688.3	1112.9	0.068 +/− 0.025	0.025	0.134	37.5	198.4
5	3-Carene	756.1	1144.2	0.082 +/− 0.072	0.010	0.371	11.9	453.1
6	β-Myrcene	795.3	1162.3	0.188 +/− 0.086	0.079	0.614	42.2	326.4
7	D-Limonene	893.6	1204.9	0.453 +/− 0.178	0.181	1.050	40.0	231.9
8	Eucalyptol	916.4	1211.8	0.403 +/− 0.446	0.000	1.442	0.0	357.8
9	β-Phellandrene	921.7	1213.4	0.627 +/− 0.889	0.000	3.678	0.0	586.7
10	trans-β-Ocimene	1003.9	1238.2	2.040 +/− 1.082	0.477	5.561	23.4	272.6
11	β-Ocimene	1057.7	1254.4	1.572 +/− 1.464	0.048	6.102	3.1	388.2
12	3-Octanone	1065.7	1256.9	1.108 +/− 0.723	0.000	2.878	0.0	259.7
13	Hexyl acetate	1124.2	1274.5	0.285 +/− 0.117	0.093	0.566	32.5	198.7
14	3-Octanol, acetate	1337.2	1338.8	0.070 +/− 0.075	0.000	0.395	0.0	566.4
15	1-Octen-3-yl-acetate	1489.7	1384.8	0.828 +/− 0.468	0.069	2.016	8.3	243.4
16	1-Octen-3-ol	1742.7	1457.0	0.190 +/− 0.132	0.035	0.618	18.4	324.8
17	Camphor	1965.3	1519.6	0.088 +/− 0.039	0.037	0.232	42.4	263.3
18	Linalool	2096.0	1556.4	24.788 +/− 8.364	7.457	45.286	30.1	182.7
19	Linalyl acetate	2130.8	1566.2	44.435 +/− 9.455	19.247	61.662	43.3	138.8
20	α-Santalene	2168.2	1576.7	0.387 +/− 0.345	0.000	1.511	0.0	390.1
21	Borneol acetate	2198.9	1585.4	0.176 +/− 0.119	0.000	0.534	0.0	302.7
22	α-Bergamotene	2209.6	1588.4	0.080 +/− 0.073	0.000	0.312	0.0	388.2
23	β-Caryophyllene	2248.0	1599.2	4.739 +/− 2.819	1.457	16.888	30.7	356.3
24	Lavandulyl acetate	2299.1	1614.4	2.739 +/− 2.158	0.170	11.249	6.2	410.7
25	α-Humulene	2496.7	1673.3	0.991 +/− 1.581	0.000	6.266	0.0	632.6
26	Crypton	2503.6	1675.4	0.433 +/− 0.384	0.000	1.129	0.0	260.6
27	Lavandulol	2540.6	1686.4	0.148 +/− 0.153	0.000	0.987	0.0	668.9
28	Borneol	2614.7	1708.5	0.512 +/− 0.266	0.016	1.443	3.2	281.6
29	γ-Cadinene	2793.5	1761.9	0.226 +/− 0.188	0.004	0.801	1.9	355.1
30	p-Isopropylbenzaldehyde	2880.7	1787.9	0.200 +/− 0.051	0.099	0.313	49.6	156.0
31	Caryophyllene oxide	3293.1	1994.5	0.408 +/− 0.205	0.127	1.274	31.2	312.6
32	p-Cymen-7-ol	3406.2	2123.2	0.299 +/− 0.134	0.054	1.232	18.2	412.4
33	tau.-Cadinol	3465.8	2194.7	0.953 +/− 0.634	0.153	2.910	16.0	305.2
34	Coumarin	3791.1	2510.7	0.590 +/− 0.219	0.186	1.308	31.6	221.8

* Average amount for the entire population and minimal and maximal amounts of the volatile compound in the flower extracts from the plants of the segregating population. ** Calculated ratios of the minimal and maximal amount scored for the compound to the average amount of the compound for the entire population.

**Table 5:** Overview of detected QTLs associated with volatile terpene contents.

No	Trait	QTL Name	LOD *	LG	Peak (cM)	Left Marker	Right Marker	PVE (%) **	Additive (%) ***
1	Toluene	q01-5.1	5.27	5	2.00	ABIL527A	ABIL103	25.9	−0.083
2		q01-5.2	5.26	5	95.82	ABIL531	ABIL164	25.6	−0.075
3	Camphene	q02-11.1	4.80	11	81.94	ABIL416B	ABIL77	23.9	−0.064
4		q02-20.1	4.58	20	48.52	ABIL505	ABIL15	22.6	−0.025
5	β-Pinene	q03-11.1	5.35	11	86.12	ABIL416B	ABIL77	26.2	−0.024
6	Sabinene	q04-3.1	4.04	3	54.74	ABIL506	ABIL456A	20.1	−0.019
7		q04-3.2	4.79	3	61.19	ABIL322	ABIL63	23.8	−0.022
8		q04-19.1	4.17	19	38.07	E10M8-344	ABIL429	21.1	0.015
9	3-Carene	q05-5.1	15.61	5	38.45	ABIL330	E4M1-85	58.8	0.083
10	D-Limonene	q06-19.1	5.43	19	32.53	ABIL11	E10M8-344	26.6	0.121
11	β-Phellandrene	q07-2.1	4.67	2	38.20	ABIL473	ABIL530A	23.3	−0.563
12		q07-19.1	4.81	19	31.53	ABIL11	E10M8-344	23.9	0.605
13	trans-β-Ocimene	q08-2.1	4.45	2	14.08	ABIL163	ABIL519	22.3	−0.644
14		q08-2.2	4.03	2	49.02	ABIL91B	ABIL268	20.5	−0.660
15		q08-6.1	6.96	6	13.00	ABIL517A	ABIL538	32.7	0.811
16		q08-24.1	4.09	24	33.42	ABIL393B	ABIL585	20.7	−1.042
17	3-Octanone	q09-1.1	12.20	1	6.96	ABIL510	ABIL14	50.0	0.681
18		q09-1.2	6.24	1	52.47	ABIL388	ABIL361	29.8	0.565
19		q09-1.3	4.49	1	106.83	ABIL371	ABIL352	22.8	0.471
20	3-Octanol acetate	q10-1.1	6.20	1	17.72	ABIL14	ABIL512	29.7	0.057
21	1-Octen-3-yl-acetate	q11-4.1	4.10	4	128.91	ABIL323	ABIL245	20.8	0.317
22		q11-8.1	6.03	8	33.40	ABIL550	ABIL548	29.0	0.206
23	1-Octen-3-ol	q12-8.1	4.39	8	59.13	ABIL554A	ABIL408	22.1	0.105
24	Linalool	q13-6.1	4.33	6	21.09	ABIL538	ABIL66	21.8	−5.217
25		q13-8.1	12.17	8	34.01	ABIL550	ABIL548	50.2	9.134
26	Linalyl acetate	q14-8.1	11.95	8	33.40	ABIL550	ABIL548	49.3	−10.576
27	α-Santalene	q15-12.1	4.04	12	165.01	ABIL409	E2M10-214	20.5	0.384
28	Borneol acetate	q16-11.1	4.86	11	82.96	ABIL416B	ABIL77	24.2	−0.092
29	α-Bergamotene	q17-12.1	4.44	12	165.01	ABIL409	E2M10-214	22.8	0.048
30	β-Caryophyllene	q18-6.1	5.46	6	61.64	ABIL251	ABIL539A	27.4	2.180
31		q18-12.1	6.39	12	101.13	ABIL210	ABIL48	30.5	1.878
32		q18-12.2	5.41	12	168.01	ABIL409	E2M10-214	26.5	1.859
33	Lavandulyl acetate	q19-3.1	17.35	3	84.29	ABIL312	ABIL27	62.7	−2.187
34	α-Humulene	q20-6.1	5.30	6	19.09	ABIL538	ABIL66	26.0	1.053
35		q20-12.1	7.26	12	82.97	ABIL61A	ABIL560	33.8	−1.262
36		q20-12.2	8.15	12	125.52	ABIL332	ABIL187	37.1	−1.252
37	Lavandulol	q21-3.1	8.47	3	84.29	ABIL312	ABIL27	38.2	−0.135
38		q21-8.1	6.16	8	16.05	ABIL552	ABIL339	29.5	0.122
39	Borneol	q22-11.1	3.50 *	11	82.96	ABIL416B	ABIL77	18.1	−0.179
40		q22-20.1	4.50	20	44.52	ABIL505	ABIL15	22.6	−0.034
41	γ-Cadinene	q23-5.1	11.58	5	58.69	ABIL112	ABIL503B	48.2	−0.175
42	p-Isopropylbenzaldehyde	q24-6.1	4.65	6	25.09	ABIL538	ABIL66	21.3	0.035
43	tau.-Cadinol	q25-5.1	11.67	5	58.69	ABIL112	ABIL503B	48.5	−0.594

* QTLs with an LOD score higher than the LOD threshold calculated for the linkage group 11 but lower than the genome-wide LOD threshold calculated for this compound. ** the percentage of the variance explained by the QTL. *** the estimated additive effect for the QTL.

**Table 6:** Chromosome locations of QTLs and candidate genes.

Chromosome	Trait (QTL Name)	QTL Markers and Candidate Gene(s)	Chromosome Locations, kBp	Comment References
CH11	Camphene (q02-11.1) β-Pinene (q03-11.1) Borneol acetate (q16-11.1) Borneol (q22-11.1)	Left QTL marker ABIL416B	30,426	*LaBPPS (L. angustifolia* bornyl diphosphate synthase) LaBPPS catalyzes the production of various by-products, including borneol, camphene, and β-pinene [25]. BLAST search with sequence acc. number KM015221.
		*LaBPPS-like*	30,794 30,802 30,819 30,831 30,834 30,841 30,855 30,872 30,880 30,922	
		Right QTL marker ABIL77	31,003	
CH12	α-Santalene (q15-12.1) α-Bergamotene (q17-12.1) β-Caryophyllene (q18-12.2)	Left QTL marker ABIL409	26,385	LaBERS (*L. angustifolia* trans-alpha-bergamotene synthase) LaBERS catalyzes the production of various by-products including α-santalene, α-bergamotene, and β-caryophyllene [68]. BLAST search with sequence acc. number DQ263742
		*LaBERS-like*	28,836 28,848 30,868 30,899	
		Right QTL marker E2M10-214	SRAP marker	
CH5	γ-Cadinene (q23-5.1) tau.-Cadinol (q25-5.1)	Left QTL marker ABIL112	29,285	LaCADS (*L. angustifolia* tau-cadinol synthase) LaCADS catalyzes the production of various by-products, including γ-cadinene and tau.-cadinol [69]. BLAST search with sequence acc. number JX401282
		*LaCADS-like*	30,139 30,143 30,154 30,158	
		Right QTL marker ABIL503B	32,864	
CH8	Linalool (q13-8.1) Linalyl acetate (q14-8.1)	Left QTL marker ABIL550	20,261	LiATT4 (*Lavandula × intermedia* alcohol acetyltransferase) [70]. LiATT4 catalyzes the production of linalyl acetate from linalool as substrate [67]. BLAST search with sequence acc. number KM275344.
		*LiAAT4*	20,389	
		Right QTL marker ABIL548	20,883	
CH6	Linalool (q13-6.1)	Left QTL marker ABIL538	27	LaLINS (*L. angustifolia* linalool synthase) LaLINS catalyzes the production of linalool from geranyl diphosphate [68]. BLAST search with sequence acc. number DQ263741
		*LaLINS*	362	
		Right QTL marker ABIL66	548	
CH5	3-Carene (q05-5.1)	Left QTL marker ABIL330	15,474	Li3CARS (*L. × intermedia* 3-carene synthase) Li3CARS catalyzes the production of 3-carene [71]. BLAST search with sequence acc. number KX024762
		*Li3CARS*	26,547 26,570 26,605	
		Right QTL marker E4M1-85/ABIL13	28,143	

## Data Availability

Raw NGS sequences can be accessed at the NCBI Sequence Read Archive under project number PRJNA1207064.

## Supplementary Materials

The following supporting information can be downloaded at: https://www.mdpi.com/article/10.3390/ijms26083705/s1.

## Abbreviations

The following abbreviations are used in this manuscript:

SSR	simple sequence repeat
SRAP	sequence-related amplified polymorphism
QTL	quantitative trait locus
GC/MS	gas chromatography/mass spectrometry
